# One Drop | Mobile: An Evaluation of Hemoglobin A1c Improvement Linked to App Engagement

**DOI:** 10.2196/diabetes.8039

**Published:** 2017-08-24

**Authors:** Chandra Y Osborn, Joost R van Ginkel, David Rodbard, Mark Heyman, David G Marrero, Brian Huddleston, Jeff Dachis

**Affiliations:** 1 Informed Data Systems, Inc New York, NY United States; 2 Leiden University Leiden Netherlands; 3 Biomedical Informatics Consultants LLC Potomac, MD United States; 4 University of California, San Diego San Diego, CA United States; 5 The University of Arizona Health Sciences Tucson, AZ United States

**Keywords:** type 1 diabetes, type 2 diabetes, mobile app, tracking, self-care, glycemic control

## Abstract

**Background:**

Three recent reviews evaluated 19 studies testing the hemoglobin A1c (HbA1c) benefit of 16 diabetes apps, including 5 publicly available apps. Most studies relied on small samples and did not link app engagement with outcomes.

**Objective:**

This study assessed both HbA1c change in a large sample of people using the One Drop | Mobile app and associations between app engagement and changes in HbA1c.

**Methods:**

The One Drop | Mobile app for iOS and Android is designed to manually and passively (via Apple HealthKit, Google Fit, and the One Drop | Chrome blood glucose meter) store, track, and share data. Users can schedule medication reminders, view statistics, set goals, track health outcomes, and get data-driven insights. In June 2017, we queried data on people with diabetes using the app who had entered at least 2 HbA1c values in the app >60 and ≤365 days apart. Multiple imputation corrected for missing data. Unadjusted and adjusted mixed effects repeated measures models tested mean HbA1c change by time, diabetes type, and their interaction. Multiple regression models assessed relationships between using the app to track food, activity, blood glucose, and medications and HbA1c change.

**Results:**

The sample (N=1288) included people with type 1 diabetes (T1D) (n=367) or type 2 diabetes (T2D) (n=921) who were 35% female, diagnosed with diabetes for a mean 9.4 (SD 9.9) years, and tracked an average 1646.1 (SD 3621.9) self-care activities in One Drop | Mobile between their first (mean 8.14% [SD 2.06%]) and second HbA1c entry (mean 6.98% [SD 1.1%]). HbA1c values were significantly associated with user-entered average blood glucose 90 days before the second HbA1c entry (rho=.73 to .75, *P*<.001). HbA1c decreased by an absolute 1.07% (unadjusted and adjusted F=292.03, *P*<.001) from first to second HbA1c entry. There was a significant interaction between diabetes type and HbA1c. Both groups significantly improved, but users with T2D had a greater HbA1c decrease over time than users with T1D (F=10.54, *P*<.001). For users with T2D (n=921), HbA1c decreased by an absolute 1.27% (F=364.50, *P*<.001) from first to second HbA1c entry. Finally, using One Drop | Mobile to record food was associated with greater HbA1c reductions even after adjusting for covariates and after also adjusting for insulin use for users with T2D (all *P*<.05).

**Conclusions:**

People with T1D and T2D reported a 1.07% to 1.27% absolute reduction in HbA1c during a median 4 months of using the One Drop | Mobile app. Using the app to track self-care was associated with improved HbA1c. More research is needed on the health benefits of publicly available diabetes apps, particularly studies associating app engagement with short- and long-term effects.

## Introduction

There are over 1500 mobile apps in the marketplace assisting with diabetes self-management but limited research on their clinical benefit. In the past year, a handful of systematic reviews and meta-analyses evaluated the impact of diabetes apps on glycemic control or hemoglobin A_1c_ (HbA_1c_) [1-4]. Three reviews included a total of 19 studies evaluating 16 unique apps. Only 5 of those apps were publicly available (ie, dBees [5], Diabeo [6], Glucose Buddy [7], mDiab/Mobil Diab [8,9], and WellDoc [10,11]).

The trials evaluating publicly available apps offer insights into their clinical value. For example, people with type 1 diabetes (T1D) using the dBees self-care and glucose tracking app had no HbA_1c_ improvement over time or compared to people tracking with a paper logbook [5]. Children and adolescents with T1D using mDiab/Mobil Diab [8] lowered their HbA_1c_, but not significantly more than a conventional care control group. In contrast, people with type 2 diabetes (T2D) using mDiab/Mobil Diab lowered their HbA_1c_ significantly more than the usual care control group [9]. In 2 separate randomized controlled trials (RCTs), people with T1D using the Diabeo insulin dosing app [6] or the Glucose Buddy tracking app [7] lowered their HbA_1c_ significantly more than controls did. Finally, people with T2D using the WellDoc tracking and coaching app substantially lowered their HbA_1c_ relative to controls [10,11].

Limited clinical evidence supporting publicly available diabetes apps is promising, but there are still many unknowns. In the 7 trials reporting data, no studied sample was greater than 200 people, which has implications for generalizability. Moreover, effects on glycemic control were linked to being exposed to an entire intervention or app and not using the app or different aspects of it.

Qualitative studies indicate people with diabetes (PWD) want apps with automated self-care tracking [12], medication reminders [13,14], data sharing with peers and providers [15] including reports [16], and a Bluetooth-connected meter [17]. Publicly available apps offer these and other features (eg, One Drop | Mobile), but studies linking engagement with such features to health outcomes are limited.

Additional studies are needed to broaden generalizability by testing with larger samples and associating app engagement to health outcomes. To address these gaps, we assessed HbA_1c_ changes among a large sample of people with T1D and T2D (N=1288) using the One Drop | Mobile app. We also assessed if using the app resulted in significant changes in glycemic control as measured by HbA_1c_ values.

## Methods

### One Drop | Mobile

The One Drop | Mobile app was launched in April 2015. It is available for free on iOS, WatchOS, and Android operating systems.

**Figure 1:**
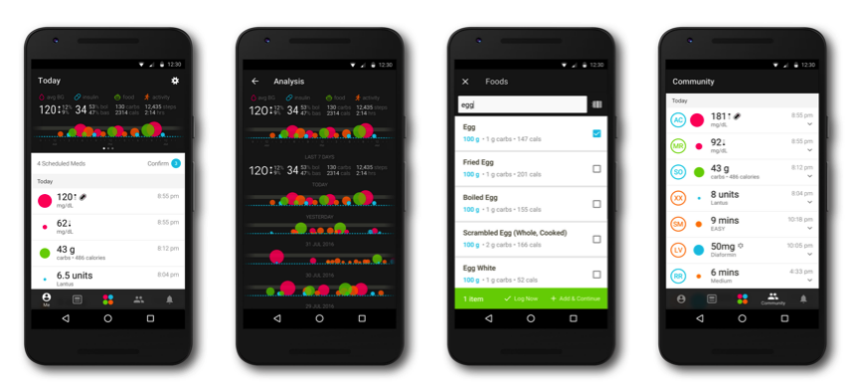
The One Drop | Mobile app.

One Drop | Mobile has a variety of features to support diabetes management (see Figure 1). Users can manually and passively (via HealthKit, Google Fit, and the Bluetooth-enabled One Drop | Chrome blood glucose meter) store and track blood glucose readings, medication doses, physical activity, and foods consumed. In addition, users can view daily, weekly, and monthly summary statistics regarding these data. A built-in food library facilitates tracking food. An optional medication scheduler reminds users when a dose is due and facilitates tracking medications. Users can also view the percentage of in-range blood glucose readings over time and store and track HbA_1c_ values and body weight. Importantly, they can set daily goals (for time in range, medication adherence, carbohydrate intake, and physical activity) and monitor their progress toward these goals. Users can also access a wide array of diabetes-relevant information by using an in-app newsfeed that delivers health tips, articles, infographics, user polls, expert interviews, and scientific study results. A community section lets the user view and learn from other users’ data. A map displays dots representing other One Drop | Mobile users in a local area, anywhere, and provides an option to view another user’s data and give badges to offer support and encouragement. A notifications inbox delivers data-driven insights, achievements, reminders, and lists badges accumulated from other users.

### Measures

#### User Characteristics

All One Drop | Mobile users complete a profile and can self-report gender, diabetes type, and year of diagnosis. We calculated years of diagnosed diabetes as the difference between a user’s year of diagnosis entered in the app and the year his or her One Drop | Mobile profile was created. We used passively collected time zone data to determine user location. Because few users outside the United States had entered 2 HbA_1c_ values required for inclusion, we dichotomized location to United States versus outside the United States in analyses.

#### Self-Care

One Drop | Mobile users can track blood glucose, medication, and physical activity manually and passively (via HealthKit, Google Fit, and the Bluetooth-enabled One Drop | Chrome blood glucose meter) in the app. They can also track their food consumed (measured in grams of carbohydrate). We summed data tracked between HbA_1c_ entries to obtain counts of blood glucose, medications, activity, and food tracked during that time.

#### Glycemic Control

Users can also self-report HbA_1c_ test results and test dates. HbA_1c_ values can be displayed in mmol/mol or percent but are stored as percent. Shortly after a One Drop | Mobile account is created, an in-app pop-up asks each user to enter his or her HbA_1c_ information. This reminder appears again 3 months after the previously entered HbA_1c_ test date. We used HbA_1c_ test dates to calculate the number of days between HbA_1c_ entries and converted days to months using the factor 30.42 (365 days/12 months.

### Study Oversight

Solutions Institutional Review Board approved analyses and reporting of One Drop | Mobile’s data for research purposes.

### Analyses

Summary statistics characterized the sample overall and stratified by diabetes type. Distributions of continuous variables were asymmetrical, so Mann Whitney U tests compared mean ranks of continuous user characteristics, app-tracked data, and HbA_1c_ percent. Chi-square tests assessed differences in dichotomous variables by diabetes type. To examine and exclude invalid self-reported blood glucose and HbA_1c_ data, we converted each user’s 90-day average blood glucose to an estimated HbA_1c_ using the formula HbA_1c_=(90-day mean blood glucose+77.3)/35.6 [18]. We calculated the difference between the converted HbA_1c_ and self-reported HbA_1c_ and excluded users with more than a 2.0% difference. For the remaining users, Spearman’s rho correlations tested the relationship between self-reported HbA_1c_ values and the prior 90-day average blood glucose to ensure consistency with the literature [19]. Because most users enter their first HbA_1c_ when they initiate using the app, we were unable to assess the relationship between 90-day average blood glucose prior to the first self-reported HbA_1c_.

Missing data were handled using multiple imputation [20]. We used predictive mean matching (PMM) [21,22] to impute 100 datasets. PMM is a multiple-imputation method robust to violations of distributional assumptions (eg, normality) [23,24]. Multiple imputation was carried out in SPSS 23.0 (IBM Corp).

Next, 3 mixed effects repeated measures models tested mean HbA_1c_ change by time (pre- to posttest), diabetes type (T1D vs T2D), and their interaction. Only these effects were in the first model (ie, the unadjusted model). The second model adjusted for a priori covariates: gender, location (US vs non-US), years since a diagnosis of diabetes, and the number of months between the first and second HbA_1c_ entries. We restricted the third model to users with T2D, excluded the time by diabetes type interaction term, and adjusted for gender, location, years since diagnosis, number of months between HbA_1c_ entries, and insulin use.

Finally, 4 multiple regression models assessed the relationships between change in HbA_1c_ and using the app to track blood glucose, activity, medications, and food. The first, unadjusted model assessed the relationships between HbA_1c_ change and the 4 types of self-care tracking. The second model included diabetes type (T1D vs T2D), and the third model included gender, location, years since diagnosis, and number of months between the first and second HbA_1c_ entries. We restricted the fourth model to users with T2D and included insulin use as well as the a priori covariates. Given the skewness of self-care data and assumption violations for statistical testing, we dichotomized each variable to indicated tracking or nontracking of blood glucose, medications, activity, and food.

## Results

As of June 6, 2017, 2365 One Drop | Mobile users had entered 2 HbA_1c_ values into the app at least 60 days but no more than 1 year apart. They reported a diagnosis of T2D (1526/2365, 64.5%), T1D (591/2365, 25%), prediabetes (122/2365, 5.2%), latent autoimmune diabetes in adults (LADA) (72/2365, 3.0%), gestational diabetes (9/2365, 0.4%), other types of diabetes (eg, surgically or chemically induced diabetes; 29/2365, 1.2%), or did not enter a diabetes type (16/2365, 0.7%).

We restricted analyses to users reporting a diagnosis of T1D or T2D and confirmed the diagnosis through examination of the names of diabetes medications logged or scheduled in One Drop | Mobile. A total of 408 T1D or T2D users were excluded from the sample because they had either no medication data or because the medications logged or scheduled were inconsistent with their stated diabetes type (eg, T1D on metformin or sulfonylurea, T2D setting an auto basal insulin).

We excluded an additional 288 users with >2.0% HbA_1c_ difference between their second self-reported HbA_1c_ and the HbA_1c_ calculated from their 90-day mean blood glucose. This criterion resulted in correlations of rho=.75 and rho=.73 between the 90-day mean blood glucose and second self-reported HbA_1c_ for subjects with T1D (n=367) and T2D (n=921), respectively (both *P*<.001). This is consistent with previous cohort studies reporting correlations between average blood glucose and HbA_1c_ varying from 0.71 to 0.86 [19].

Three of the up to 14 variables included in analyses had missing data: gender (242/1288, 18.8%), location (14/1288, 1.1%), and duration of diagnosed diabetes (325/1288, 25.5%). Multiple imputation was used to make corrections for missing data on these variables.

Analyses included N=1288 users (see Table 1) who were 35% (454/1288) female, diagnosed with diabetes for a mean 9.4 (SD 9.9) years, and tracked an average 1646.1 (SD 3621.9) self-care activities in One Drop | Mobile between their first (mean 8.14% [SD 2.06%]) and second (mean 6.98% [SD 1.1%]) HbA_1c_ (calculations prior to multiple imputation).

Table 1 presents preimputed median and interquartile range (IQR) or n (%) with *P* values for differences between diabetes type on app-entered user characteristics, app-tracked data, and HbA_1c_ entries. Chi-square tests compared dichotomous variables. Mann Whitney U tests compared mean ranks of continuous variables in Table 1.

**Table 1:** Sample characteristics with tests of difference by diabetes type.

Characteristics		Total N=1288	Type 1 diabetes n *=* 367	Type 2 diabetes n=921	*P* value
**Gender, n (%)**					
	Male	592 (46.0)	154 (42.0)	438 (47.6)	.61
	Female	454 (35.2)	152 (41.4)	302 (32.8)	
	Other	2 (0.2)	1 (0.3)	1 (0.1)	
**Location, n (%)**					
	America/United States	1077 (83.6)	292 (80.7)	785 (86.1)	.001
	Europe	111 (8.6)	51 (14.1)	60 (6.6)	
	Asia	44 (3.4)	7 (1.9)	37 (4.1)	
	Pacific	16 (1.2)	4 (1.1)	1.3 (14)	
	Australia	19 (1.5)	5 (1.4)	1.5 (3)	
	Africa	6 (0.5)	3 (0.8)	3 (0.3)	
	Atlantic	1 (0.1)	0 (0.0)	1 (0.1)	
**Insulin, n (%)**					
	Yes	717 (55.7)	367 (100)	350 (38)	.001
Diabetes duration in years, median (IQR)		6 (15)	10 (19)	5 (12)	.001
Food entries, n (%)		4 (88)	10 (99)	3 (82)	.04
Activity entries, n (%)		271.5 (809)	182 (786)	294 (814)	.09
Blood glucose entries, n (%)		72 (200)	102 (356)	67 (165)	.001
Medication entries, n (%)		118.5 (366)	121 (609)	117 (331)	.02
Months between HbA_1c_ entries, median (IQR)		4.0 (3.1)	4.6 (1.5)	3.9 (2.7)	.001
First HbA_1c_ %, median (IQR)		7.6 (2.4)	7.65 (2.1)	7.6 (2.5)	.43
Second HbA_1c_ %, median (IQR)		6.9 (1.4)	7.30 (1.5)	6.7 (1.3)	.001

Compared to users with T2D (367/1288), users with T1D (921/1288) were diagnosed with diabetes for more years (*U*_958_= 71,571, *z*=–7.07, *P*<.001), had more months between their first and second HbA_1c_ (for both *U*_1286_=140,143.5, *z*=–4.79, *P*<.001), and tracked more food (*U*_1286_=156,703.5, *z*=–2.09, *P*=.04), blood glucose (*U*_1286_=147,630, *z*=–3.56, *P*<.001), and medications (*U*_1286_=155,500, *z*=–2.26, *P*=.02). They were also more likely than users with T2D to log or schedule insulin in the app (χ^2^_1,N=1288_=408.7, *P*<.001), use the app in Europe (χ^2^_1,N=1274_=24.1, *P*<.001), and report a higher second HbA_1c_ (*U*_1286_=125,966.5, *z*=–7.14, *P*<.001).

**Figure 2:**
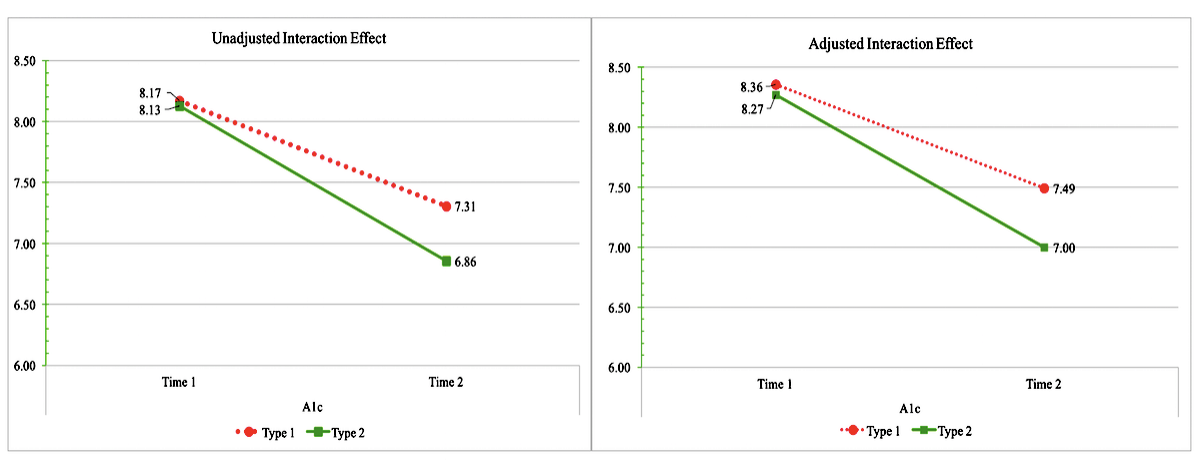
The unadjusted and adjusted interaction between diabetes type and hemoglobin A1c over time.

In the unadjusted model (Multimedia Appendix 1), HbA_1c_ decreased by an absolute 1.07% (*F*=292.03, *P*<.001) in the median 4.0 (IQR 3.1) months from first (mean HbA_1c_ 8.15%) to second entry (mean 7.08%). Users with T1D (mean 7.74%) had an absolute .25% (*F*=9.52, *P*=.002) higher HbA_1c_ than users with T2D (mean 7.49%). There was a significant interaction between diabetes type and HbA_1c_ entry (Figure 2). Both groups improved over time, but users with T2D had a greater HbA_1c_ decrease over time than users with T1D (*F*=10.54, *P*<.001).

After adjusting for gender, location, duration of diabetes, and months between HbA_1c_ entries, HbA_1c_ continued to decrease by an absolute 1.07% (*F*=292.03, *P*<.001; Multimedia Appendix 1) from first (mean HbA_1c_ 8.31%) to second entry (mean HbA_1c_ 7.24%). Regardless of time, users with T1D (mean 7.92%) continued to have a higher HbA_1c_ (.29% HbA_1c_ difference; *F*=11.66, *P*<.001) than users with T2D (mean 7.63%). In the adjusted model, the interaction between diabetes type and HbA_1c_ entry persisted (Figure 2). Users with T2D continued to have a greater HbA_1c_ decrease over time than users with T1D (*F*=10.54, *P*<.001). After adjusting for gender, location, duration of diabetes, months between HbA_1c_ entries, and insulin use, users with T2D reported a 1.27% absolute HbA_1c_ reduction (*F*=364.43, *P*<.001) from first (mean HbA_1c_ 8.16%) to second entry (mean HbA_1c_ 6.89%).

Finally, using the app to record food was associated with greater HbA_1c_ reductions even after adjusting for covariates and after further adjusting for insulin use for users with T2D (Multimedia Appendix 2, *P*<.05).

## Discussion

### Principal Findings

We assessed changes in HbA_1c_ for people with T1D or T2D who used the One Drop | Mobile app over a period of 1 year. We also evaluated relationships between tracking self-care with the app and HbA_1c_ change during that time. App users reported up to a 1.27% absolute decrease in HbA_1c_ depending on their diabetes type. Using the app to track food intake was associated with greater HbA_1c_ reductions.

Landmark studies, including the Diabetes Control and Complications Trial [25] and United Kingdom Prospective Diabetes Study [26], found lowering HbA_1c_ closer to normal levels reduced the risk of diabetes complications. According to recent reviews, diabetes apps are associated with reduction of HbA_1c_ [1-3], but their effectiveness varies widely across studies and by diabetes type.

One review reported people with T1D who used diabetes apps had a 0.36% HbA_1c_ reduction in 3 to 9 months [1]. For people with T1D using the dBees self-care and blood glucose tracking app, there was no HbA_1c_ reduction over time or relative to controls using a paper logbook [5]. In another trial, 34 children and teenagers with T1D using the mDiab/Mobil Diab tracking and self-care support app reduced their HbA_1c_ by 0.72%, but HbA_1c_ also fell by 0.98% in the control group [8]. In a nonrandomized controlled trial, 90 adults with T1D and HbA_1c_≥8% using the Diabeo digital diary and insulin calculator lowered their HbA_1c_ by 0.91% relative to controls [6]. Among 36 people with T1D in Australia using the Glucose Buddy tracking app, HbA_1c_ was reduced by 1.10% [7].

Based on 367 people with T1D using the One Drop | Mobile app, we found HbA_1c_ declined by 0.86%—an amount consistent with other studies evaluating publicly available apps but more than two-fold larger than the overall effect of diabetes apps tested among people with T1D [1]. Moreover, unlike the previous trials described above, we related HbA_1c_ change to tracking self-care with an app, and found, regardless of diabetes type, using the app to track food consumption was associated with a greater HbA_1c_ reduction.

For people with T2D, an evaluation of 10 studies testing diabetes apps found an average HbA_1c_ reduction of 0.49% [3]. One of those studies was an RCT evaluating the publicly available WellDoc app (now available as Bluestar) that reported a 2.03% drop in HbA_1c_ among 15 people with T2D in one urban area. Our observational study with no control group or randomization included a sample of 921 people with T2D, and found HbA_1c_ decreased by 1.27%. This HbA_1c_ improvement is comparable to the difference in HbA_1c_ improvement between the WellDoc intervention and control groups and more than double the effect of diabetes apps used by people with T2D in a recent meta-analysis by Hou et al [3]. In that meta-analysis, one other trial evaluated a publicly available app [9]. The trial evaluated the mDiab/Mobil Diab app as used by 40 people with T2D in Butembo, Democratic Republic of Congo [9]. HbA_1c_ improved by 1.78% [9]. The baseline HbA_1c_ was 0.54% higher than in our study.

### Limitations

This study has limitations. There was no control group or randomization. Multiple potential confounding factors may have contributed to the observed results, making it impossible to ascribe causal relationships between using the One Drop | Mobile app and HbA_1c_ change. The significant relationship between using the app to track self-care and HbA_1c_ benefit enhances confidence of a direct link. Users were also self-selected in terms of their using the app and self-reporting 2 or more HbA_1c_ values, introducing external validity and generalizability concerns. This possibility, however, is also a concern with any RCT in which participants self-select to participate. Our sample also reflects people willing to use a diabetes app. It is plausible to assume these people are younger, have a higher socioeconomic status (ie, a higher income, education) and are more comfortable using technology. To protect privacy, One Drop | Mobile does not collect user age, precluding the ability to describe this and other characteristics (eg, education, income, insurance status) of the sample or adjust for them in analyses. One Drop | Mobile also has other features we did not relate to HbA_1c_ change or adjust for in our analyses. HbA_1c_ was self-reported rather than assessed with a laboratory assay. Because the app is a tool for the user and not subject to review by others, it is unlikely users altered their HbA_1c_ values in response to social desirability bias. Consistent with prior studies that used laboratory HbA_1c_ values, we found a greater HbA_1c_ improvement among people with T2D than people with T1D [1]. Also, self-reported HbA_1c_ was highly correlated with average blood glucose 90 days before the HbA_1c_, increasing confidence in its utility as an indicator of glycemic control in this study. Finally, our sample included over 1200 PWD from both within and outside the United States, differentiating it from other studies that included people from only one country or region.

### Conclusion

There are currently no best practices for evaluating mobile health apps [27], and clearly more research is needed. This study adds to that body of work. Diabetes app developers collect data that can both improve product offerings and user experience and evaluate how users may be benefiting.

We believe people want and deserve mobile health apps that address their self-care needs and enhance their ability to improve the management of their chronic health condition [17,28]. Selecting an app is challenging. There are over 1500 diabetes apps to choose from with more being developed. A review of 65 publicly available diabetes apps concluded 86% were unfit for promoting self-management [29]. Ratings by consumers can be a poor indication of an app’s clinical efficacy [30]. The results of carefully developed clinical evaluations will help consumers select better apps and assist providers in recommending efficacious apps to patients.

## Appendix

Multimedia Appendix 1 Tests of mean hemoglobin A_1c_ change by time, diabetes type, and their interaction.

Multimedia Appendix 2 Tests of the relationships between tracking food, activity, blood glucose, and medications in One Drop | Mobile and hemoglobin A1c change.

## Abbreviations

HbA _1c_: hemoglobin A_1c_
IQR: interquartile range
LADA: latent autoimmune diabetes in adults
PMM: predictive mean matching
PWD: people with diabetes
RCT: randomized controlled trial
T1D: type 1 diabetes
T2D: type 2 diabetes
