# Supplementary material for: One Drop | Mobile: An Evaluation of Hemoglobin A1c Improvement Linked to App Engagement
Source: JMIR Diabetes. 2017 Aug 24;2(2):e21. doi: 10.2196/diabetes.8039 (PMC6238886; doi:10.2196/diabetes.8039)
Supplement: Multimedia Appendix 1 [file diabetes_v2i2e21_app1.pdf]

Multimedia Appendix 1. Tests of mean hemoglobin A_{1c} change by time, diabetes type, and their interaction.

	Users with Type 1 or Type 2 Diabetes (*N* = 1,288)										Users with Type 2 Diabetes (*n* = 921)				
	Unadjusted					Adjusted					Adjusted				
	Est.	Std. Error	F	p-value	95% CI	Est.	Std. Error	F	p-value	95% CI	Est.	Std. Error	F	p-value	95% CI
Intercept	7.62	.04	36130.4	.001	(7.54, 7.69)	7.78	.10	6068.4	.001	(7.58, 7.98)	7.52	.11	4348.1	.001	(7.30, 7.74)
Time	.53	.03	292.0	.001	(.47, .60)	.53	.03	292.0	.001	(.47, .59)	.64	.03	362.5	.001	(.57, .70)
Diabetes type	.12	.04	9.5	.002	(.05, .20)	.15	.04	11.7	.001	(.06, .23)	---	---	---	---	---
Time * Diabetes type	-.10	.03	10.5	.001	(-.16, -.04)	-.10	.03	10.6	.001	(-.16, -.04)	---	---	---	---	---
Gender						-.08	.04	3.5	.061	(-.16, .00)	-.08	.05	2.6	.118	(-.17, .02)
Location						.09	.05	3.1	.076	(-.01, .19)	.05	.06	.6	.454	(-.07, .16)
Diabetes duration in years						-.00	.00	.7	.405	(-.01, .01)	-.01	.01	4.1	.043	(-.03, -.00)
Months between A1c entries						-.03	.01	5.6	.018	(-.06, -.01)	-.04	.02	5.4	.020	(-.08, -.01)
Insulin											.66	.10	45.8	.001	(.47, .85)

Note. Est. = effect estimates, CI = confidence interval. Results of mixed effects repeated measures models are presented.
