# Supplementary material for: One Drop | Mobile: An Evaluation of Hemoglobin A1c Improvement Linked to App Engagement
Source: JMIR Diabetes. 2017 Aug 24;2(2):e21. doi: 10.2196/diabetes.8039 (PMC6238886; doi:10.2196/diabetes.8039)
Supplement: Multimedia Appendix 2 [file diabetes_v2i2e21_app2.pdf]

Multimedia Appendix 2. Tests of the relationships between tracking food, activity, blood glucose, and medications in One Drop | Mobile and A1c change.

	Users with Type 1 or Type 2 Diabetes (N = 1,288)															Users with Type 2 Diabetes (n = 921)				
	Unadjusted					Adjusted for Diabetes Type					Adjusted for Covariates					Adjusted for Covariates				
	B	Std. Error	F	p-value	95% CI	B	Std. Error	F	p-value	95% CI	B	Std. Error	F	p-value	95% CI	B	Std. Error	F	p-value	95% CI
Food entries	.39	.15	6.4	.012	(.09, .69)	.44	.15	8.2	.004	(.14, .74)	.51	.15	11.6	.001	(.22, .81)	.50	.17	8.9	.003	(.17, .82)
Activity entries	.26	.23	1.3	.26	(-.17, .68)	.23	.23	.96	.326	(-.15, .60)	.28	.22	1.6	.209	(-.16, .72)	-.09	.27	10.9	.741	(-.63, .45)
Blood glucose entries	-.37	.29	1.7	.202	(-.66, .08)	-.44	.29	2.3	.130	(-.99, .11)	-.60	.28	4.4	.036	(-1.15, -.04)	-.14	.34	0.2	.672	(-.80, .52)
Medication entries	-.22	.23	.94	.333	(-.67, .23)	-.20	.23	.74	.391	(-.64, .25)	-.24	.22	1.1	.288	(-.64, .20)	-.13	.25	0.3	.600	(-.61, .35)
Diabetes type						.44	.13	12.3	.001	(.20, .69)	.05	.13	.18	.675	(-.20, .31)	---	---	---	---	---
Gender											.38	.13	9.0	.003	(.13, .62)	.41	.14	8.1	.004	(.13, .69)
Location											.19	.15	1.6	.207	(-.11, .50)	.10	.19	.30	.583	(-.27, .47)
Diabetes duration in years											-.05	.01	60.7	.001	(-.06, -.04)	-.07	.01	45.8	.001	(-.09, -.05)
Months between A1c entries											-.08	.02	13.0	.001	(-.13, -.04)	-.12	.03	17.5	.001	(-.17, -.06)
Insulin																.39	.15	6.9	.008	(.10, .68)

Note. B = unadjusted coefficient, CI = confidence interval. Results of multiple regression models are presented.
